# The Kallisti Limnes, carbon dioxide-accumulating subsea pools

**DOI:** 10.1038/srep12152

**Published:** 2015-07-16

**Authors:** Richard Camilli, Paraskevi Nomikou, Javier Escartín, Pere Ridao, Angelos Mallios, Stephanos P. Kilias, Ariadne Argyraki, and the Caldera Science Team, Muriel Andreani, Valerie Ballu, Ricard Campos, Christine Deplus, Taoufic Gabsi, Rafael Garcia, Nuno Gracias, Natàlia Hurtós, Lluis Magí, Catherine Mével, Manuel Moreira, Narcís Palomeras, Olivier Pot, David Ribas, Lorraine Ruzié, Dimitris Sakellariou

**Affiliations:** 1 Woods Hole Oceanographic Institution, Department of Applied Ocean Physics and Engineering, Woods Hole, MA, 02543, USA; 2 University of Athens, Faculty of Geology and Geoenvironment, Panepistimiopoli Zographou, 15784 Athens, Greece; 3 Institut de Physique du Globe de Paris, CNRS UMR7154, 75238 Paris Cedex 05, France; 4 University of Girona, Computer Vision and Robotics Group, 17071 Girona, Spain; 5 Institute of Oceanography, Hellenic Centre for Marine Research, Anavyssos, Greece; 6 Laboratoire de Géologie de Lyon, UMR 5276, ENS et Université Lyon 1, 69622 Villeurbanne Cedex, France; 7 University of Manchester, School of Earth, Atmospheric and Environmental Science, Oxford Road Manchester M13 9PL: UK

## Abstract

Natural CO_2_ releases from shallow marine hydrothermal vents are assumed to mix into the water column, and not accumulate into stratified seafloor pools. We present newly discovered shallow subsea pools located within the Santorini volcanic caldera of the Southern Aegean Sea, Greece, that accumulate CO_2_ emissions from geologic reservoirs. This type of hydrothermal seafloor pool, containing highly concentrated CO_2_, provides direct evidence of shallow benthic CO_2_ accumulations originating from sub-seafloor releases. Samples taken from within these acidic pools are devoid of calcifying organisms, and channel structures among the pools indicate gravity driven flow, suggesting that seafloor release of CO_2_ at this site may preferentially impact benthic ecosystems. These naturally occurring seafloor pools may provide a diagnostic indicator of incipient volcanic activity and can serve as an analog for studying CO_2_ leakage and benthic accumulations from subsea carbon capture and storage sites.

Anthropogenic carbon dioxide (CO_2_) inputs and naturally occurring marine hydrothermal venting can profoundly impact marine food webs and ecosystems^1^,^2^,^3^. Sub-seafloor storage of CO_2_ within geological formations is gaining international acceptance as a mechanism for reducing CO_2_ emissions and lessening the impacts of ocean acidification^4^,^5^. However, specific concerns over leakage of CO_2_ from sub-seafloor injection sites necessitate field studies in order to understand associated risks^6^,^7^.

The Santorini volcanic group and Kolumbo submarine volcano are located along the Hellenic Volcanic Arc in the southern Aegean^8^,^9^. The prehistoric Minoan eruption of the Santorini caldera, circa 1627-1600 B.C.^10^, was one of the largest in human history, with a total eruption volume of up to 60 km^3^^,^^8^. This eruption buried prehistoric settlements, including the flourishing Bronze Age city of Akrotiri^10^,^11^. Subsequent volcanic eruptions formed the Kameni islands, which emerged in 197 B.C. at the center of the caldera. Historical records from the past five centuries reveal a characteristic interval of 61 ± 68 years between eruptions, with the latest eruption occurring in 1950^12^.

In January 2011, following more than six decades of quiescence, the Santorini caldera entered a renewed phase of unrest characterized by sustained seismicity, caldera-wide inflation, and increased gas emissions. Spherical Mogi models of deformation from January 2011 to April 2012 suggest a source centered 1.5 km north of Nea Kameni, 4 km beneath the caldera’s Northern Basin^13^ (Fig. 1), and a corresponding volume increase of 1 to 2 × 10^7^ m^3^ 14,15. Isotopic analysis of magmatic and thermal decarbonation gas emissions indicate increasing mantle CO_2_ contribution^16^. These variations coincide with episodic charging of its shallow magma chamber by high-flux batches of deeper melts which historically control small effusive and large explosive eruptions^15^.

On July 21, 2012 our research team explored the North Basin using an experimental autonomous underwater vehicle (AUV) to survey this area of the crater (Supplementary Fig. S1). In addition to engineering demonstration operations, our deployment objectives included better characterization of the caldera’s subsea regions of activity to aid the Santorini archipelago’s hazard preparedness.

## Results

The two initial AUV survey operations were conducted at an altitude of between 5 and 20 meters above the seafloor. The first dive was completed in one hour and the second in approximately 1.5 hours, with 2.5 hours elapsing between the dives. In-situ data recorded by AUV’s payload sensors indicated a water column interval between −270 and −230 m depth containing temperature, methane, and carbon dioxide anomalies (Fig. 2). Based on these geochemical signals, we launched a close inspection survey with a human occupied submersible vehicle (HOV) to investigate the seafloor in close proximity (altitude of less than 1 meter). The HOV survey commenced two hours after completion of the second ROV dive at a previously-identified hydrothermal field^8^,^9^ in the North Basin. Northeast of this hydrothermal site we encountered small (0.1 to 1 m diameter) mounds of unconsolidated flocculent orange-colored microbial mats extending up the steep NE caldera wall at a depth range of −300 to −250 m. In these shallower depths the flocculent mats are larger and denser than mounds located deeper within the basin. In steep areas along the caldera wall, linear ridges and flow channels develop within meter-thick orange flocculent microbial mats and Fe-rich clastic sediments. These channels are less than 1 m deep and up to 2 m wide, with NE-SW down slope trending lengths in excess of 20 m (Fig. 3). Upslope of the flow channels, between −250 and −235 m depth, and within 1 km of the cliff-top town of Oia, we discovered an interconnected series of meandering, iridescent white pools (Fig. 3). These 1 to 5 m diameter pools, named *Kallisti Limnes (translation from Ancient Greek: Most Beautiful Lakes)*, are situated within localized depressions in terraced scallops of the slope wall. Unlike other sites of known CO_2_ venting^16^ along the Christianna-Santorini-Kolumbo (CSK) tectonic line^17^, ebullition was not observed at the Kallisti Limnes and visual inspection yielded no evidence of flow movement or percolation.

The recessed locations of the Kallisti Limnes pools did not permit sampling of their fluids with the HOV. However, in-situ mass spectrometer measurements recorded during the HOV survey of the ambient seawater one meter above the pools indicate pCO_2_, dissolved methane, and dissolved oxygen concentrations ranging from 490 to 547 μatm, 28 to 44 nM, and 130 to 131 μM, respectively. The dissolved oxygen concentrations are consistent with values found at this depth in this region of the Aegean^18^, but the pCO_2_ concentrations were substantially elevated compared to Aegean waters at this temperature^19^, and the methane concentrations were similar to levels previously measured along the lines of known fracture zones outside the caldera^20^, suggesting that the pools are associated with hydrothermal activity. Therefore, a follow-up investigation was conducted to sample the pools’ fluids using a remotely operated vehicle (ROV) equipped with a Niskin bottle sampler and temperature probe (Supplementary Fig. S2).

A one-hour time series recorded by the temperature probe while deployed within the pool fluids on July 22, 2012 indicates a stable elevated temperature of 21.5 °C, or approximately 5.5 °C greater than the surrounding ambient seawater at this depth (Supplementary Figs. S3 and S4). Ex-situ analysis of the pool fluids collected using the ROV Niskin sample bottles revealed a pH of 5.93, 41 PSU salinity (2 PSU greater than the ambient seawater at this depth) along with pCO_2_, dissolved methane, and dissolved oxygen concentrations of 50,400 μatm, 30 nM, and 80 μM, respectively. The pool fluid’s concentrations represent a pCO_2_ enrichment approximately 100 fold greater than the adjacent seawater, and nearly identical dissolved methane concentration. The pool fluid’s relatively minor dissolved oxygen decrease of 39% suggests, however, that it contained a mixture of seawater and hydrothermal fluids, and may substantially under-represent the CO_2_ enrichment of the hydrothermal fluids.

Mats and suspended particulate material from the Kallisti Limnes are composed of iron oxyhydroxide ooze with biogenic silica debris (diatoms and other siliceous shells), minor phases such as kaolinite, glass and pumice volcanic debris, and trace amounts of K-feldspar and chlorite, but devoid of carbonate minerals. Microscopic examination of the material revealed filamentous Fe-rich and spherical Si-rich structures (Fig. 4).

## Discussion

**Figure 1:**
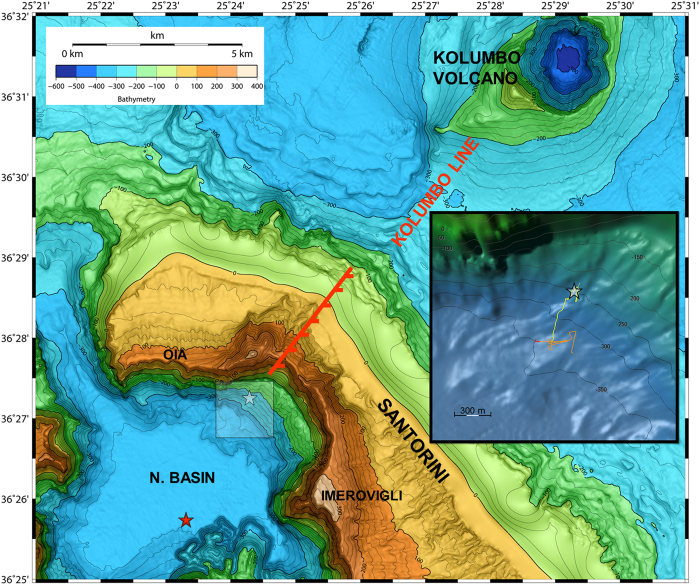
Topographic relief map of the northern Santorini volcanic field. A red star icon shows the location of the center of inflation deduced from InSAR data^15^, the white star icon shows the location of the Kallisti Limnes CO_2_ pools. The onshore Kolumbo fault is indicated by a dashed red line, which along with the Kolumbo line, describes the northeast portion of the Christianna-Santorini-Kolumbo (CSK) tectonic line. The inset box shows a detailed view from the southwest of the caldera slope bathymetry around the study site; submersible vehicle track lines are indicated as red, orange, and yellow lines, corresponding to the first and second AUV dives, and the HOV dive, respectively. Sonar and vehicle position data was processed using MB—System Revision 1.15 (including source code derived from other sources, described at http://www.ldeo.columbia.edu/res/pi/MB-System/html/mbsystem_copyright.html), GMT-4.5.7, MATLAB, and Fledermaus version 7.4.1 c software.

The Kallisti Limnes and vent mounds are in line with the Kolumbo normal fault onshore (Kolumbo Line in Fig. 1), which belongs to the CSK tectonic line^17^. This tectono-volcanic fracture zone is of predominantly right-lateral transtensional character, which extends from the Christianna volcanic group northeast to the Kolumbo volcanic chain and likely controls hydrothermal circulation pathways within the caldera. The pools and flow channels that form the Kallisti Limnes are in close proximity to a shallow magmatic intrusion within the sediments of the Santorini North Basin^15^. The locations and thermal and chemical composition of these pools suggest that they are a locus of fluid seepage from the CSK tectonic line.

The high Si content of mats (Supplementary Table S1) and suspended particulate material from the Kallisti Limnes is consistent with amorphous opal (~10 wt%), generating the pools’ iridescent coloration, while high Fe content (Supplementary Table S1) associated with oxyhydroxides is indicative of a rapid upward flux of Fe(II)-rich waters derived either from sub-seafloor hydrothermal processes^20^ and/or connate waters from Fe-rich sediments^21^. Outward flow of reduced Fe-rich sub-seafloor fluids is further supported by high concentrations of free Fe measured within the orange microbial mats. Low S concentration (Supplementary Table S1) and the absence of sulfide and sulfate minerals do not indicate precipitation from brine. Rare earth element distributions (Supplementary Fig. S5) exhibit negative chondrite-normalized Eu anomalies, which are consistent with low-temperature (<100 °C) acidic fluids (pH < 4)^22^, and/or subseafloor reaction of felsic crustal rocks with low pH acid–sulfate fluids^23^. Eu/Eu*, La/Yb, and Zr/Fe ratios (Supplementary Table S1) indicate bio-detrital derived particulate matter^24^.

The Fe-oxyhydroxide particles in the mats generally appear as biomorphous fine-grained aggregates with helical ribbon-like morphologies (Fig. 4) resembling the stalk of known Fe-oxidizing bacteria *Gallionella ferruginea* (previously isolated from hydrothermal sediments within the Santorini Caldera^25^), and/or *Mariprofundus ferrooxydans*^26^,^27^. Environmental clones within this group of Fe-oxidizing organisms have been obtained from CO_2_-rich/H_2_S-poor hydrothermal vent systems^26^, and Fe–Si-rich precipitates in deep-sea low-temperature hydrothermal environments^27^. The dense microbial mats associated with these pools indicate that fluid seepage has persisted on a time scale sufficient to allow extensive colonization and the meandering flow channels within the microbial mats provide clear evidence of persistent low velocity, gravity-driven flow. Their inactivity during our field investigations further suggests a temporally varying flow regime.

Pycnoclinic seafloor pools exist throughout the world, including the Eastern Mediterranean^28^,^29^, and are characteristically generated through dissolution of upward injected evaporites into younger, surficial seafloor sediments^30^. Although the salinity content of the Kallisti Limnes is only slightly elevated and not sufficient to be considered as brines, the massively elevated carbon dioxide content of the Kallisti Limnes increases the fluid density by at least 82 to 109 g·m^−3^. Under sufficiently low energy (i.e., thermal and dispersive mixing) conditions this density increase may be adequate for stratification and gravity-driven flow^31^.

The process leading to the formation of the Kallisti Limnes and their elevated CO_2_ content remains uncertain. Localized regions of microbial mats were observed cascading downward in areas of the slope walls directly above the highest elevation hydrothermal pool. These microbial mats appeared to initiate as point sources along specific geologic strata, providing indirect evidence of dense fluid sources seeping at low velocities along flow conduits within the volcano-sedimentary strata. Alternatively, if gaseous carbon dioxide venting occurs from the seafloor, it should in principle generate buoyancy, causing CO_2_ to be driven upward through the water column. The rapid dissolution of CO_2_ gas into the water column may nonetheless increase the entrained water’s density to such a degree that it separates into a gravity-driven plume convecting CO_2_-laden water downward^7^,^31^,^32^. Previous investigations of gaseous CO_2_ venting within the nearby Kolumbo volcano identified dilute acidified benthic waters within its crater, but localized pools of CO_2_ accumulations were not observed^33^.

**Figure 2:**
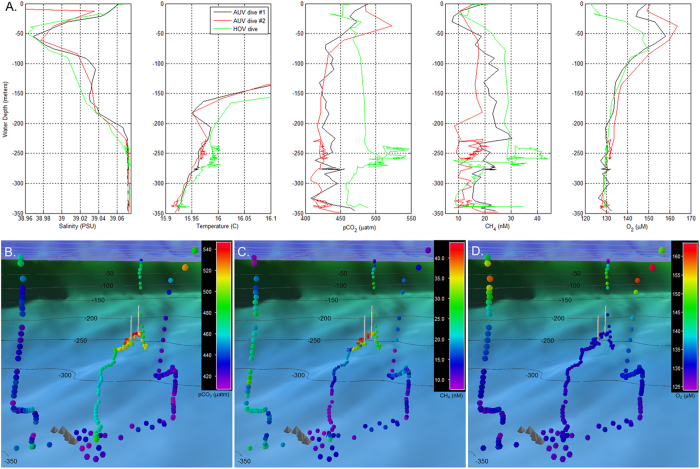
Water column profile data recorded during AUV and HOV survey operations. **A**. Water column profiles of salinity, temperature, carbon dioxide partial pressure, and dissolved methane, and dissolved oxygen distributions recorded during two initial AUV reconnaissance dives and the subsequent HOV investigation dive. Anomalies are visible in the 270 to 230 meter depth range. **B., C**. and **D**. show geo-referenced pCO_2_, methane, and dissolved oxygen distributions respectively, recorded during the AUV and HOV dive missions. Colored circles indicate dissolved chemical concentrations with corresponding color bar key located in the upper right. Chemical measurements are shown as viewed from the Santorini North Basin, above the center of inflation, looking toward the Kolumbo volcano. Gray pyramid-shaped icons indicate locations of hydrothermal vent mounds identified within the North Basin during prior ROV dive operations; white vertical lines indicate the uppermost and lowermost locations of the Kallisti Limnes on the caldera wall. The dark blue color field with gray grid in the upper portion of **B**., **C**., and **D**. indicates the sea surface.

**Figure 3:**
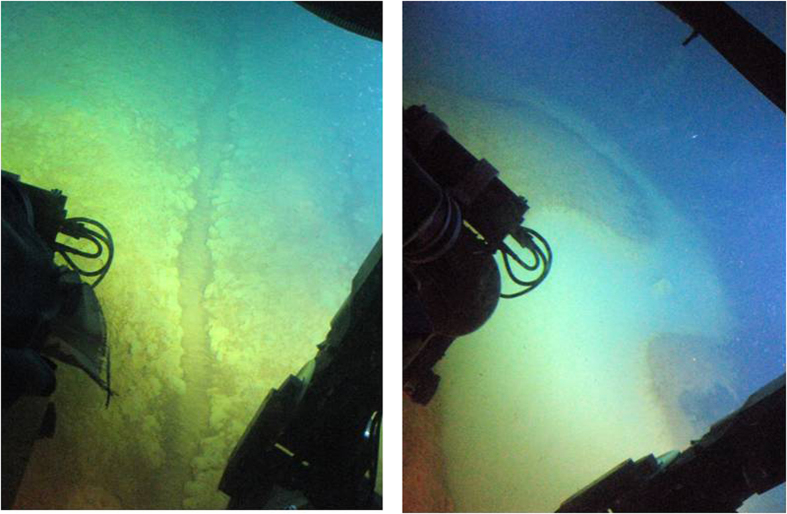
Carbon dioxide-accumulating subsea pools. Left, photo of dense microbial Fe-mats with a narrow, 0.1 m wide, flow channel cascading down slope from a pool at −250 m depth. Right, photo at −235 m depth showing a 1–2 m wide meandering Kallisti Limnes hydrothermal pool (image recorded at a 45° rotation to maximize field of view). The characteristic meandering of these pools and flow channels suggest slow, persistent gravity-driven flow.

**Figure 4:**
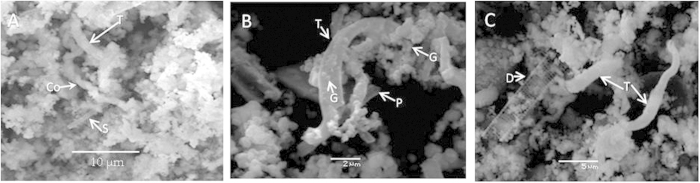
Scanning Electron Microscope (SEM) images of Si-Fe-rich particles from mats and suspended particulate materials. Morphologies are consistent with microbially produced structures by iron-oxidizing genera: **A**. Coiled (Co), curved (T) and slender sheath-like (S), filaments; helical or twisted structures resemble the stalks of *Gallionella ferruginea or Mariprofundus ferrooxydans*^26^,^27^. **B**. Irregularly twisted branching filamentous (T), and platy (P), forms; spheroid cell-like grains (G) commonly precipitate on the surface of filaments. **C**. Curved non-coiled sheath-like septated structures (S); diatom frustule (D) with striae are also visible.

The Kallisti Limnes represent a previously unobserved but theoretically contemplated marine phenomenon: concentrated seafloor CO_2_ accumulations generated by venting from a geologic reservoir^33^,^34^. The pools are situated below the basin’s sill depth of 150 m, which presumably controls the outflow of the CO_2_-rich basin water, but enrichments of dissolved methane and pCO_2_ were detected in the water column directly above the pools to a depth of 25 m, indicating transport into surface waters (Fig. 2A,B,C). Although the CO_2_ concentrations measured during this investigation have shown that the pool fluids are under-saturated with respect to CO_2_ by approximately two orders of magnitude, further research is required to determine the CO_2_ inventory of these pools and if their accumulation rate can exceed mixing and carbonate speciation rates to a degree that they present an environmental hazard^34^ similar to the freshwater “killer” lakes of Cameroon^35^.

Unlike evaporite brine pools, which can persist as relatively stable seafloor features, the shallow volcanic slope setting, relatively minor total inorganic carbon (C_T_)-induced density difference, and carbonate system buffering suggest that the Kallisti Limnes may be ephemeral structures, oscillating between CO_2_ accumulation and re-emission. Their location within the caldera’s slope provides clear evidence of previously unknown hydrothermal activity in close proximity to densely populated areas on Santorini. The pools may provide a new diagnostic tool for detecting localized geophysical activity (e.g., variations in the magmatic system or hydrothermal pathways) and a natural analog for studying leakage dynamics and environmental impact from subsea floor anthropogenic carbon storage sites. Further study is required to locate the CO_2_ source of these pools and determine if CO_2_ accumulation within the Kallisti Limnes is linked to the onset of renewed volcanic unrest. In addition to monitoring seismicity and terrestrial inflation, monitoring the size and composition of these pools may aid the archipelago’s hazard preparedness by providing insight into the caldera’s subsea dynamics.

## Methods

### Submarine Reconnaissance

Multibeam bathymetric surveys were carried out by the R/V AEGAEO using a hull-mounted SEABEAM 2120 swath system operating at 20 kHz during multiple cruises in 2001–2006 and 2012. This sonar has an angular coverage sector of 150° with 149 beams, covering a swath width from 7.5 to 11.5 times the water depth for depths from 20 m to 5 km. Initial reconnaissance dives were conducted with the Girona 500^36^, a recently developed 500 m depth-rated AUV (Supplementary Fig. S1). It was equipped with conventional navigation sensors (Doppler velocity log, attitude, heading, roll, ultra-short baseline acoustic transponder, pressure, and sound velocity) as well as analytical payload sensors (profiler sonar, side scan sonar, video camera, a digital stereo still imaging system, Imagenex (DeltaT) multibeam swath microbathymetry, and TETHYS mass spectrometer^37^). AUV missions were conducted in a water column profiling mode, with the mass spectrometer autonomously recording dissolved gas distributions in-situ, principally methane (M/Z 15), dissolved oxygen (M/Z 32), and carbon dioxide (M/Z 44). The TETHYS mass spectrometer is equipped with an integrated Seabird FastCat49 conductivity, temperature, and depth (CTD) sensor. The Thetis HOV (Supplementary Fig. S1) was deployed following completion of the AUV dive missions for closer inspection of water column chemical anomalies. Thetis is a Comex Remora 2000 class HOV, capable of operating up to 610 m depth for up to nine hours mission duration. Visual survey was documented using multiple still cameras within the 2 person crew cabin (pilot and scientist). For this dive the submersible was operated in a close bottom following mode (within one meter of the seafloor) and was equipped with a TETHYS mass spectrometer and integrated Seabird FastCat49 CTD. The mass spectrometer and CTD were mounted onto the submersible’s port manipulator arm and autonomously logged the previously described chemical and physical water parameters.

### Physical sample collection and analysis

Two autonomous logging temperature probes provided temperature estimates of the Kallisti Limnes pool fluids. These sensors were installed in a plastic framework that was placed by the MaxRover remotely operated vehicle (ROV) (Supplementary Fig. S3). These temperature probes were left within the pool for one hour to allow for thermal equilibration. The instruments’ (NKE autonomous temperature sensor and WHOI-MISO low-temperature sensor) records yielded maximum temperatures within the pool of 21.5 and 19.7 °C. We report only the highest temperature in the text of this manuscript. Fluids and suspended material were collected from the Kallisti Limnes pools using 2.5 l Niskin bottles attached to the side of the ROV frame (Supplementary Fig. S2). Kallisti Limnes pool fluid samples were collected at a depth of −235 m.

Following recovery the samples’ solid phases were separated from liquid by vacuum filtration using a 0.45 μm pore filter. Solid filtrates underwent a series of geochemical analyses (ACME Labs Ltd; Vancouver, BC, Canada). Analytical portions of 0.2 g were subjected to a lithium borate fusion and dilute acid digestion. Concentrations of major elements were subsequently measured by ICP-AES while rare earths and refractory elements were measured by ICP-MS. Base metal and metalloid concentrations were analyzed by ICP-MS following an aqua regia digestion. Analytical quality was assessed by duplicate analysis and run of reference materials and was found to be within acceptable limits. Scanning Electron Microscopy was conducted on carbon-coated free surfaces of suspended particulate matter samples with a JEOL-560 instrument, equipped with an Oxford Isis 300 EDS System (National and Kapodistrian University of Athens-NKUA, Greece). The operating conditions were 20.0 kV, with a working distance of 20 mm and beam current of 0.5 nAmps.

Water sample pH was measured shipboard ex-situ, immediately following recovery of the ROV, at 23 °C using a WTW 340i, set B, pH sensor (WTW GmbH, Weilheim, Germany). The electrode was calibrated daily with three buffer solutions at pH = 4.01, 7.00 and 10.01, and was thoroughly rinsed with distilled water between each measurement. Ex-situ salinity, CO_2_, methane, and oxygen measurements recorded from Niskin water and pool fluid samples were analyzed using the TETHYS mass spectrometer and its integrated FastCAT 49 CTD (Seabird Electronics, Bellevue, Washington) which was operated in the ship’s science laboratory in a closed-loop circulation configuration. The recirculation system was thoroughly rinsed with distilled water between each measurement.

Laboratory-based TETHYS mass spectrometer calibrations for dissolved methane, oxygen, and pCO_2_ were completed prior to deployment using ultra-high purity reference gases (SCOTTY Specialty Gas Calibration Standards, Sigma-Aldrich, Saint Louis, Missouri, USA) bubbled through Milli-Q water for 15–20 min at constant temperature. Dissolved gases were then injected in a pre-evacuated temperature and pressure controlled chamber and measured using the TETHYS mass spectrometer. Each temperature calibration was carried out across a temperature span of 2 °C to 23 °C and was performed across a pressure range of 1 to 200 bar. TETHYS dissolved oxygen calibration was verified in United States coastal waters prior to the survey operations by simultaneously measurement of seawater samples using a calibrated Aanderaa Oxygen Optode 4330F (Aanderaa Data Instruments AS, Bergen, Norway).

### Estimation of pool fluid density

Ten benthic water column samples were collected using the ROV’s Niskin bottles at a depth of −340 meters in the vicinity of a previously identified hydrothermal field^8^,^9^. These water samples were collected for the purpose of establishing baseline water column chemistry. The pH of these samples was 7.924 ± 0.044, which is statistically consistent with Eastern Mediterranean intermediate waters (−700 to −200 m depth) pH = 7.962 ± 0.021^38^. In-situ salinity measurements in this baseline area was 39.07 PSU (−340 to −235 m water depth), which is also within the range of Eastern Mediterranean intermediate waters^38^. Sea water density calculations^39^ indicate that ambient water within the caldera at a depth of −235 m (15.97 °C and 39.07 PSU) has a density of 1,029,921 g·m^−3^. In contrast, the calculated density of the Kallisti Limnes pool fluids (−235 m depth, 21.5 °C, and 41.00 PSU) is 1,029,942 g·m^−3^, yielding a net density increase for the pool fluids (accounting for temperature and salinity) of 21 g·m^−3^.

Eastern Mediterranean intermediate water has a characteristic total inorganic carbon (C_T_) content of 2293 ± 15 nmol·g^−1^, with a total alkalinity (A_T_) of 2612 ± 5 nmol·g^−1 (38)^. However, if the increased salinity of the pool fluids (41 PSU) and an A_T_ -salinity relationship (80 nmol·g^−1^ per PSU^38^) are ignored in order to avoid double counting of the elevated CO_2_ density effects, a conservative estimate can be made of the pools’ C_T_ and its associated density increase. Using the ambient seawater A_T_ of 2612 and salinity of 39.07 PSU in combination with the pool fluids’ measured minimum pH of 5.93 (recorded ex-situ at 23°C) yields a C_T_ of approximately 4700 nmol·g^−1^ 40. The pool fluids’ measured pCO_2_ of 50,400 μatm (recorded ex-situ at 22.3°C), along with these ambient alkalinity and salinity values provide a second, independent C_T_ estimate of at least 4100 nmol·g^−1^ (within 13% of the pH derived value).

C_T_ calculations for the pool fluids indicate an enrichment of between 1800 and 2400 nmol·g^−1^ in excess of the ambient water column, contributing a fluid density increase of at least 82 to 109 g·m^−3^. This approximation is likely to underestimate the actual density increase because it does not take into account colloidal suspensions, such as iron and biogenic silica, or additional ions present in the pools which may also affect the fluid’s CO_2_ buffering capacity. Furthermore, the position of the Niskin bottles on the ROV prevented sampling of fluids from the bottom of the pools, making it likely that fluid samples are mixed with ambient water and under-represent the pools’ actual C_T_.

## Additional Information

**How to cite this article**: Camilli, R. *et al.* The Kallisti Limnes, carbon dioxide-accumulating subsea pools. *Sci. Rep.* **5**, 12152; doi: 10.1038/srep12152 (2015).

## Supplementary Material

Supplementary Information
