# Supplementary material for: The Kallisti Limnes, carbon dioxide-accumulating subsea pools
Source: Sci Rep. 2015 Jul 16;5:12152. doi: 10.1038/srep12152 (PMC4503996; doi:10.1038/srep12152)
Supplement: Supplementary Information [file srep12152-s1.pdf]

Title: The Kallisti Limnes, carbon dioxide-accumulating subsea pools

Richard Camilli[1*], Paraskevi Nomikou[2], Javier Escartín[3], Pere Ridao[4], Angelos Mallios[4,5], Stephanos. P. Kilias[2], & Ariadne. Argyraki[2]

and the Caldera Science Team: Muriel Andreani[6], Valerie Ballu[3], Ricard Campos[4], Christine Deplus[3], Taoufic Gabsi[4], Rafael Garcia[4], Nuno Gracias[4], Natàlia Hurtós[4], Lluis Magí[4], Catherine Mével[3], Manuel Moreira[3], Narcis Palomeras[4], Olivier Pot[3], David Ribas[4], Lorraine Ruzié[7], Dimitris Sakellariou[5]

Affiliations:

[1]Woods Hole Oceanographic Institution, Department of Applied Ocean Physics and Engineering, Woods Hole, MA, 02543, USA.

[2]University of Athens, Faculty of Geology and Geoenvironment, Panepistimiopoli Zographou, 15784 Athens, Greece.

[3] Institut de Physique du Globe de Paris, CNRS UMR7154, 75238 Paris Cedex 05, France.

[4]University of Girona, Computer Vision and Robotics Group, 17071 Girona, Spain.

[5] Institute of Oceanography, Hellenic Centre for Marine Research, Anavyssos, Greece.

[6]Laboratoire de Géologie de Lyon, UMR 5276, ENS et Université Lyon 1, 69622 Villeurbanne Cedex, France.

[7] University of Manchester, School of Earth, Atmospheric and Environmental Science, Oxford Road Manchester M13 9PL-UK.

*Correspondence and requests for materials should be addressed to RC (rcamilli@whoi.edu)

This supplement contains:

1. Supplementary Tables (1)

2. Supplementary Figures (5)

Supplementary Table 1

	CAF-16	CAF-17	CAF-19	CAF-20
Si %	16.20	16.90	12.59	
Al %	3.24	3.37	2.39	
Fe %	16.32	16.38	24.33	
Mg %	1.03	1.10	0.87	
Ca %	1.46	1.45	1.34	
Na %	4.67	4.47	2.86	
K %	0.87	0.93	0.65	
Ti %	0.17	0.18	0.13	
P %	0.07	0.09	0.05	
Mn %	0.05	0.06	0.05	
Cr	20.53	20.53	20.53	
Ni	11.00	27.00	7.60	5.8
Ba	149.00	176.00	123.00	
Co	5.40	5.30	4.40	
Cs	1.80	1.80	1.40	
Ga	5.70	5.30	3.70	
Sr	163.40	190.60	232.00	
V	94.00	118.00	86.00	
Zr	72.00	79.50	55.30	
Y	55.90	53.60	24.40	
La	12.60	13.70	9.10	
Ce	21.80	23.60	15.40	
Pr	2.62	2.78	2.01	
Nd	10.10	10.60	7.40	
Sm	2.41	2.69	1.83	
Eu	0.53	0.58	0.46	
Gd	2.93	3.03	1.96	
Tb	0.47	0.47	0.33	
Dy	3.20	3.11	2.26	
Ho	0.82	0.75	0.44	
Er	2.27	2.22	1.39	
Tm	0.37	0.36	0.22	
Yb	2.11	2.36	1.40	
Lu	0.37	0.38	0.22	
ΣREE	62.60	66.63	44.42	
La/Yb	5.97	5.81	6.50	
Eu/Eu*	0.20	0.20	0.24	
C %	1.28	1.55	1.43	1.08
S %	0.19	0.21	0.22	0.2
Mo	27.60		40.70	32.1
Cu	19.80		14.50	8.7
Pb	45.40		23.10	19.7
Zn	28.00		21.00	16
As	53.50		42.90	36.1
Zr/Fe	4.41	4.85	2.27	
As/Fe	3.28		1.76	

Table S1. Elemental concentrations (mg/kg or as otherwise stated) in suspended material of the Kallisti Limnes.

Supplementary Figures

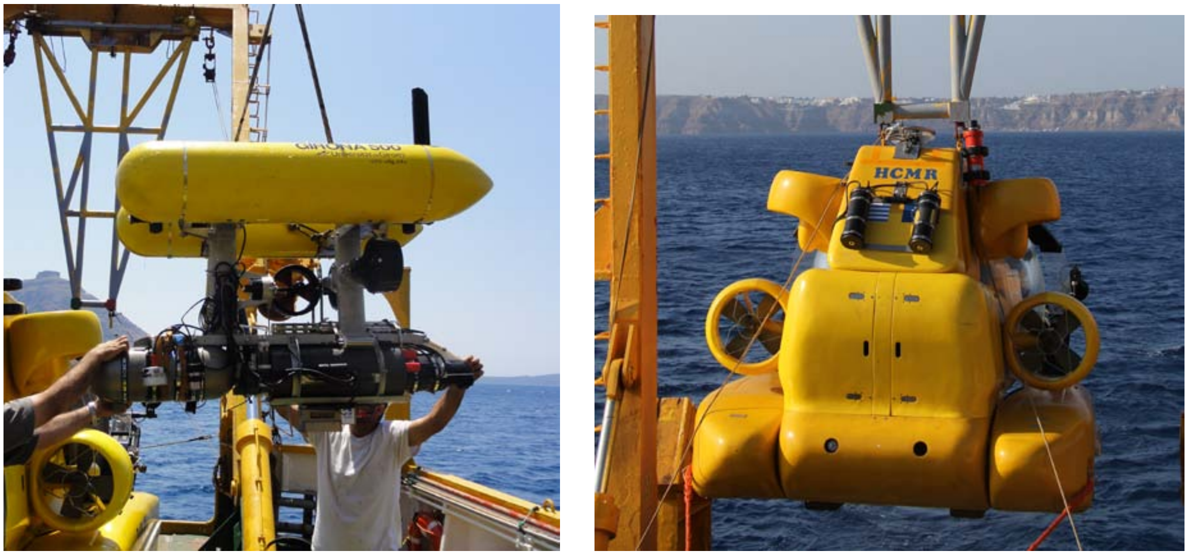

Figure S1 | Photos showing deployment of the *Girona 500* AUV and *Thetis* HOV (at left and right, respectively) equipped with a TETHYS mass spectrometer. The mass spectrometer is visible in the AUV's forward lower payload bay (left side of the photo), and its battery packs are visibly attached to the aft of the HOV (below the HCMR logo). Cliff-top towns of Santorini are visible in the distance.

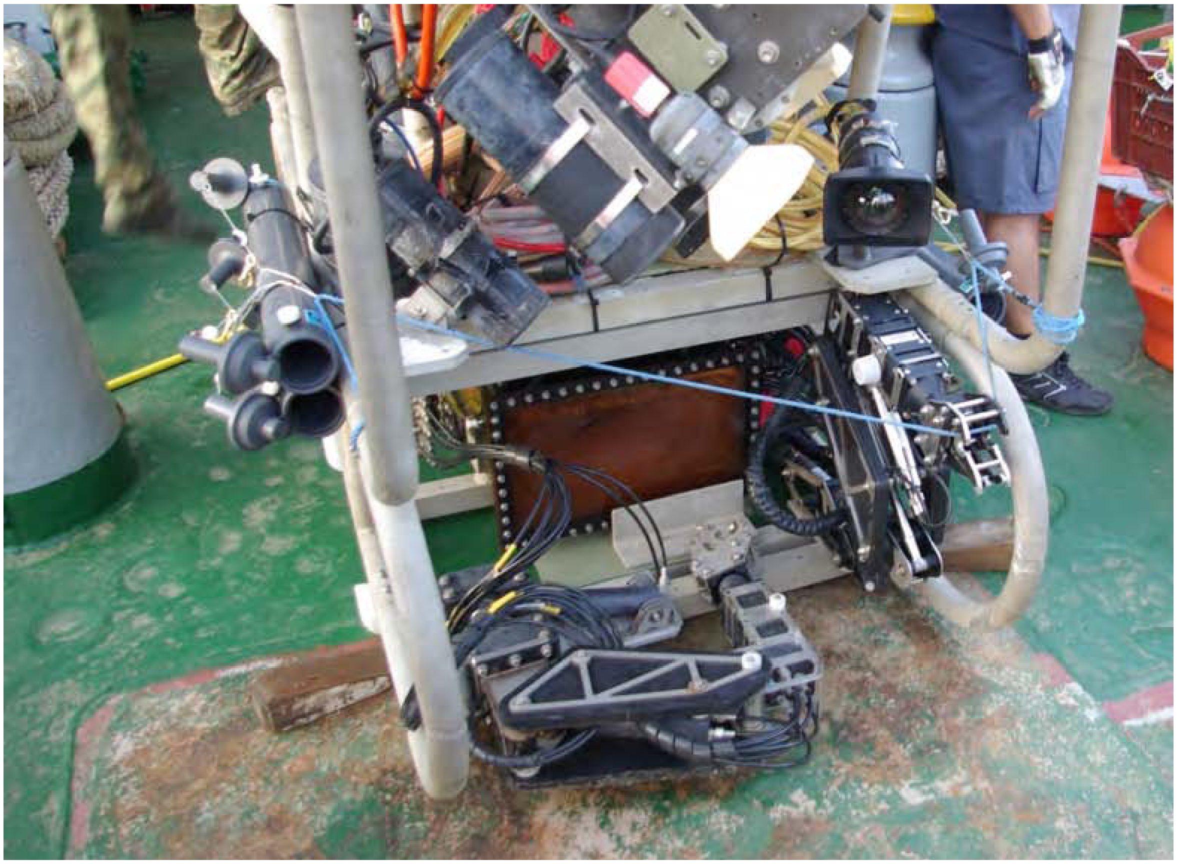

Figure S2 | Photo of the *MaxRover* ROV showing horizontally-mounted Niskin bottles attached to the port and starboard sides of the vehicle chassis approximately 0.5 meters above its bottom skids.

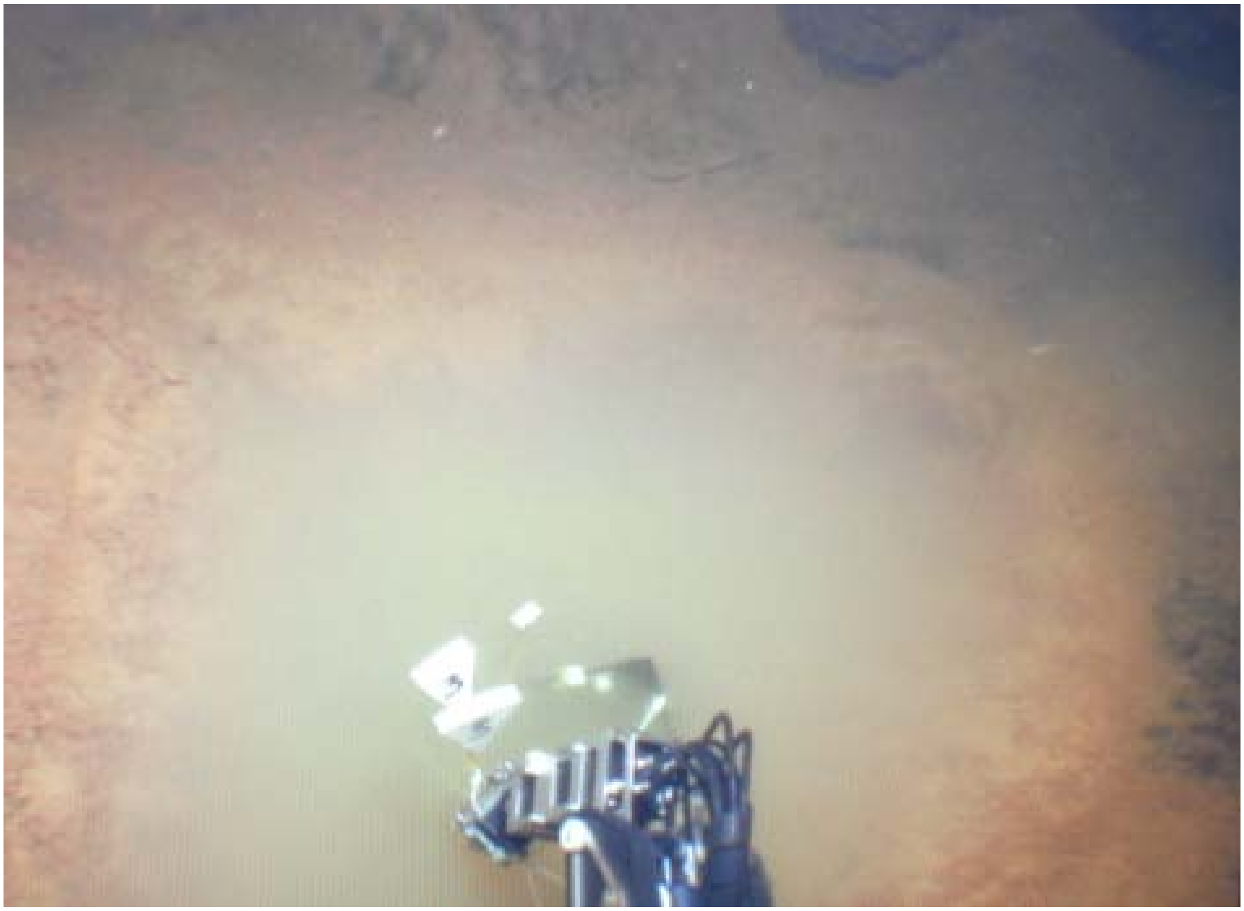

Figure S3 | Photo of the *MaxRover* ROV manipulator arm placing the temperature sensors within a Kallisi Limnes hydrothermal pool at -235 m water depth.

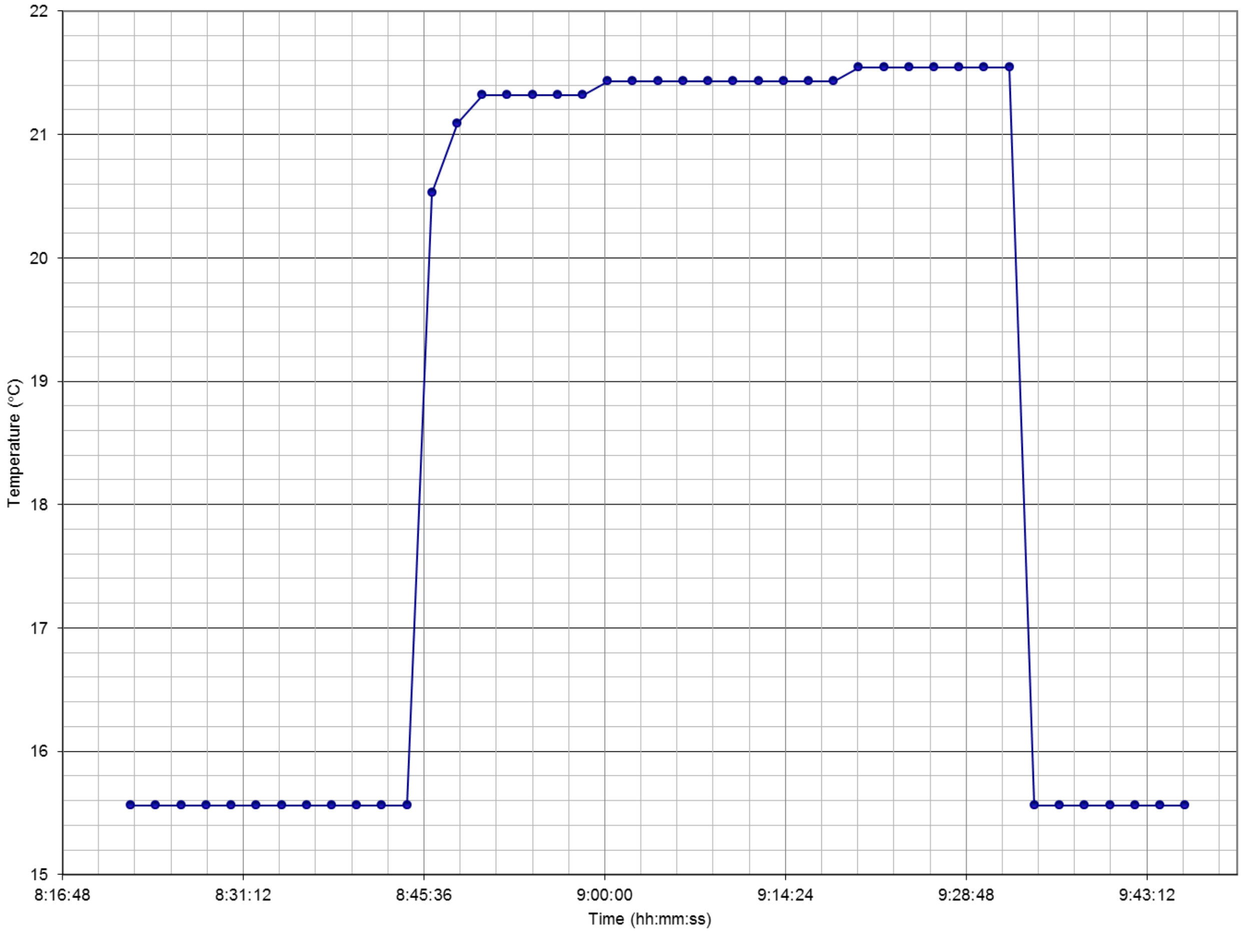

Figure S4 | One hour temperature time series recorded within the highest elevation pool of the Kallisti Limnes (depth of -235 m). Data are interpreted to represent a stable temperature within the pool, where the temperature increase from 21.319° C to 21.545° C is caused by thermal stabilization of the temperature probe. The seawater temperature immediately above the pool prior to deployment and following recovery from the pool remained unchanged at 15.563° C.

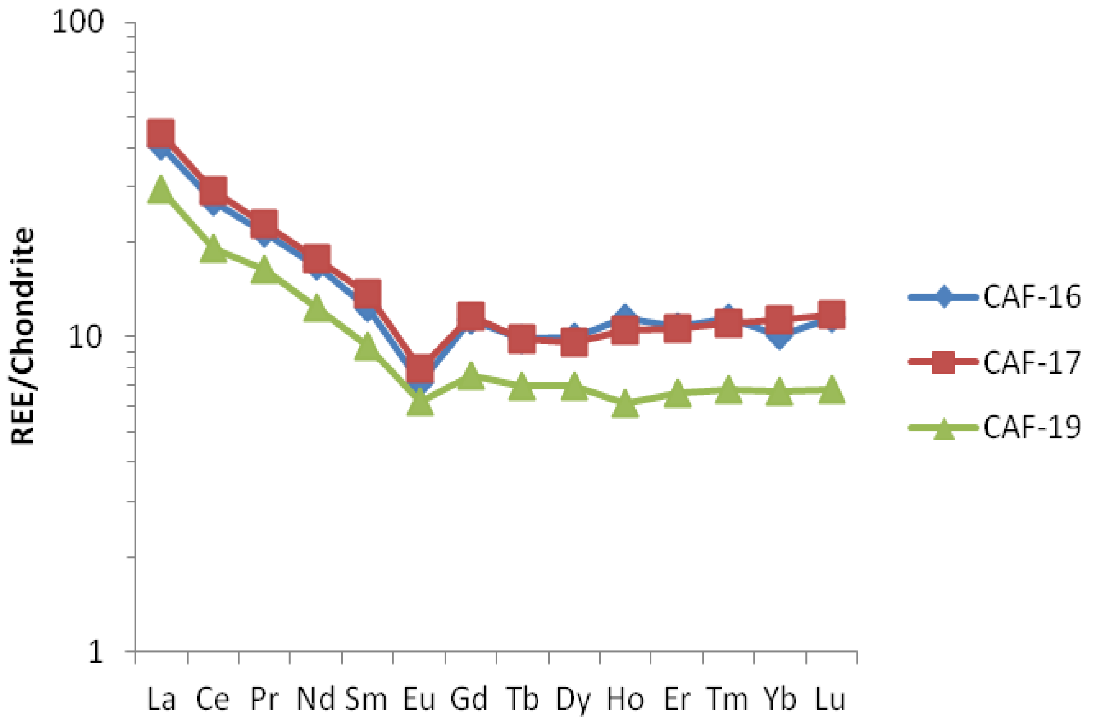

Figure S5 | Chondrite normalized rare earth element (REE) distribution patterns of the suspended material from Kallisti Limnes.
